# Determining Dynamic Mechanical Properties for Elastic Concrete Material Based on the Inversion of Spherical Wave

**DOI:** 10.3390/ma15228181

**Published:** 2022-11-17

**Authors:** Huawei Lai, Zhanjiang Wang, Liming Yang, Lili Wang, Fenghua Zhou

**Affiliations:** 1 MOE Key Lab. of Impact and Safety Engineering, Ningbo University, Ministry of Education, Ningbo 315211, China; 2 College of Geomatics and Municipal Engineering, Zhejiang University of Water Resources and Electric Power, Hangzhou 310018, China; 3 Northwest Institute of Nuclear Technology, Xi’an 710024, China

**Keywords:** spherical waves, wave propagation method, particle velocity histories, linear constitutive relation of concrete, rate-dependent

## Abstract

The paper presents a new method to study the dynamic mechanical properties of concrete under low pressure and a high strain rate via the inversion of spherical wave propagation. The dynamic parameters of rate-dependent constitutive relation of elastic concrete are determined by measured velocity histories of spherical waves. Firstly, the particle velocity time history profiles in the low stress elastic region at the radii of 100.6 mm, 120.6 mm, 140.6 mm, 160 mm, and 180.6 mm are measured in the semi-infinite space of concrete by using the mini-explosive ball and electromagnetic velocity measurement technology. Then, based on the universal spherical wave conservation equation and the fact that the accommodation relationship in state equation satisfies linear elastic law, the inverse problem analysis of spherical waves in concrete (called “NV + T0/SW”) is proposed, which can obtain the dynamic numerical constitutive behavior of concrete in three-dimensional stress by measuring the velocity histories. The numerical constitutive relation is expressed in the form of distortion, and it is found that the distortion law has an obvious rate effect. Finally, the rate-dependent dynamic parameters in concrete are determined by the standard linear solid model. The results show that the strain rate effect of concrete cannot be ignored with the strain rate range of 10^2^ 1/s. This study can provide a feasible method to determine the dynamic parameters of rate-dependent constitutive relation of concretes. This method has good applicability, especially in the study of the dynamic behavior of multicomponent composite materials with large-size particle filler.

## 1. Introduction

Concrete is widely used in engineering, and these concrete engineering facilities are often subjected to various effects, such as earthquakes, weapon strike explosions, and engineering blasting. There are usually spherical wave problems such as point explosion and point impact. Then, it is necessary to deal with the propagation of spherical waves in concrete [1,2,3]. The dynamic response or spherical wave propagation in concrete under spherical impact completely depends on the dynamic properties of the concrete. Therefore, it is important to study the dynamic properties of concrete under a high strain rate, which has attracted the attention of many researchers [4,5,6]. Bischoff et al. [4] review experimental techniques commonly used for high strain rate testing of concrete in compression and characteristics of the dynamic compressive strength and deformation behavior. Malvar and Ross [5] undertake a literature review to characterize the effects of strain rate on the tensile strength of concrete. Cusatis [6] presents a previously developed meso-scale model for concrete, including the effect of loading rate, and the rate dependence of concrete behavior is assumed to be caused by two different physical mechanisms. Some studies [7,8,9,10] indicated that the different strain-rate sensitivity is determined in concrete under different strain rates. Al-Salloum et al. [7] studied the dynamic behavior of concrete experimentally by testing annular and solid concrete specimens using a split Hopkinson pressure bar (SHPB). Wang et al. [8] designed a large-diameter SHPB with a diameter of 100 mm used to carry out impact tests at different speeds. The results show that the increase in the strain rate has a hindering effect on the increase in damage variables and the increase rate (impact speeds of 5 m/s, 10 m/s, and 15 m/s). Wang et al. [9] provided guidance for selecting pulse shapers for concrete SHPB experiments. Grote et al. [10] applied SHPB and plate impact to achieve a range of loading rates and hydrostatic pressures. 

Meanwhile, researchers have carried out many studies on rate-dependent materials of spherical waves [11,12,13,14,15,16]. Luk et al. [11] developed models for the dynamic expansion of spherical cavities from zero initial radii for elastic–plastic rate-independent materials with power-law strain hardening. Wegner et al. [12] presented a new formulation of the governing equations of spherical waves, in which the resulting system of five equations is treated as a strictly hyperbolic system of first-order hyperbolic partial differential equations, and the method of characteristics is adapted to obtain numerical solutions. Forrestal et al. [13] developed a spherical cavity-expansion penetration model for concrete targets, and predictions from the compressible penetration model are in good agreement with depth of penetration data. Lai et al. [14,15] used the ZWT linear and nonlinear visco-elastic constitutive model to set up the governing equations for linear and nonlinear visco-elastic spherical waves, and published numerical results using the characteristics method. Lu et al. [16] established the linear visco-elastic ZWT constitutive equation under a three-dimensional stress state by ignoring the relaxation effect of the low-frequency Maxwell element and the nonlinear spring element. The absorption and dispersion phenomena of the spherical wave propagation in the visco-elastic solid were analyzed. At present, with the development of experimental technology, researchers are interested in wave propagation technology (WPT) [17,18,19,20]. Zhu et al. [17] set up the error in the determination of dynamic stress–strain curve of rate-dependent brittle materials with the traditional SHPB techniques with either a three-wave method or a two-wave method, which is not accepted. Wang et al. [18] developed an experimental apparatus for spherical divergent wave propagation in solids. Liu et al. and Sollier et al. [19,20] completed a series of experiments to measure the shock initiation behavior using eleven embedded electromagnetic particle velocity gauges. The dynamic performance experiment of concrete is different from the quasi-static test. The behavior of materials under spherical impact cannot be separated from the analysis of spherical wave propagation (wave propagation effect). The core problem in carrying out this research is that the effects of wave propagation and strain rate are often coupled. When studying the dynamic constitutive relation of materials with high strain rates, the wave propagation effects in the experimental process, especially in the specimen, should not be ignored.

In order to solve the above-mentioned difficulties and deal with the coupling problem, people have developed WPT to study the dynamic properties of materials subjected to dynamic loads [21]. In various wave propagation analysis techniques, Lagrangian analysis has attracted the attention of many researchers [22,23,24,25,26], because there are no other pre-assumptions about the constitutive relation of the materials under study. In the case of spherical waves, the constitutive equation of spherical waves consists of two parts: the volumetric part and the distortional part [27]. The traditional Lagrange analysis of wave propagation is based on the conservation equations without any pre-assumption of material constitutive relation. However, when the radial particle velocity profiles are measured by velocity gauges at the Lagrangian coordinates r_i_ (i = 1,2,…), it is still difficult to solve the other two unknowns from the two constitutive equations with unknown dynamic parameters (Equations (1a), (1b), and (2)), which is different from the rate-independent elastic problem for parameters of constitutive equations, which are constant. In the work outlined in this paper, a series of particle velocity wave profiles of concrete in the far-field or low-pressure region under spherical impact loading is measured. Then, based on the universal spherical wave conservation equation and the fact that the volumetric part of constitutive relation satisfies linear elastic law, the Lagrangian inverse analysis of spherical wave problems and particle velocity history measurements (the inverse analysis) are carried out to obtain the numerical constitutive relation, expressed in the form of distortion. Furthermore, it is found that the rate-dependent characteristics of spherical wave distortion is different from the rate-independent case and therefore an appropriate rate-dependent constitutive model is chosen to describe this problem. Finally, the dynamic parameters in constitutive relation of concrete with high strain rates are obtained by the standard linear solid model.

## 2. Materials and Methods

### 2.1. Theoretical Concepts of Spherical Waves in Concrete 

Many materials have significant rate correlation characteristics under the loading of short-duration explosion and impact [28,29,30]. Concrete materials also have relevant characteristics under short-history loading [31,32,33]. The fracture strain of concrete under a high strain rate is as low as a magnitude of 10^−3^, and the behavior of concrete under one-dimensional and multidimensional stress under static load also shows great differences. Therefore, the concrete can be regarded as a linear viscose-elastic material, not just a linear elastic material.

**Figure 1:**
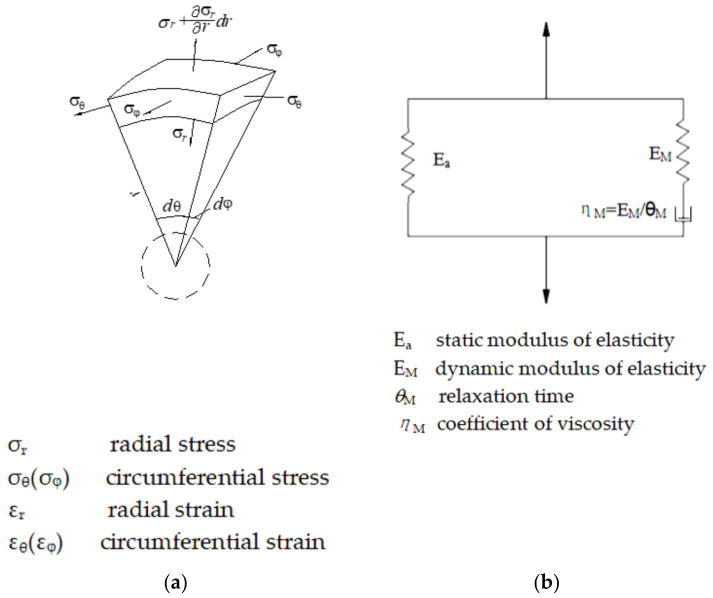
Schemes of governing equations: (**a**) micro-element in spherical coordinate system; (**b**) the standard linear solid constitutive model.

First, the description system of spherical wave propagation is established in the spherical coordinate system (Figure 1a). The governing equation system of a linear viscose-elasticity (Figure 1b) spherical wave is composed of two parts: the conservation Equations (1a) and (1b) and the constitutive Equations (2a) and (2b) (the volumetric part 2a and the distortional part 2b), representing the physical properties [34]. The linear viscose-elasticity is reflected in the distortion relation of the constitutive Equation (2b):

(1a) $$\frac{\partial \varepsilon_{r}}{\partial t}=\frac{\partial v}{\partial r},$$

(1b) $$\frac{\partial \varepsilon_{\theta }}{\partial t}=\frac{v}{r},$$

(1c) $$\frac{\partial \sigma_{r}}{\partial r}+\frac{2\left(\sigma_{r}-\sigma_{\theta }\right)}{r}=\rho_{0}\frac{\partial v}{\partial t},$$

The linear viscose-elastic constitutive equation in differential form based on the standard linear solid model can be effectively used to describe the dynamic constitutive properties of concrete (3a) [35], and Figure 1b shows how the model works.

(2a) $$\frac{\partial \sigma_{r}}{\partial t}+2\frac{\partial \sigma_{\theta }}{\partial t}-3K\left(\frac{\partial \varepsilon_{r}}{\partial t}+2\frac{\partial \varepsilon_{\theta }}{\partial t}\right)=0,$$

(2b) $$\frac{\partial \varepsilon_{r}}{\partial t}-\frac{\partial \varepsilon_{\theta }}{\partial t}=\frac{1}{2G}\left(\frac{\partial \sigma_{r}}{\partial t}-\frac{\partial \sigma_{\theta }}{\partial t}\right)+\frac{\left(\sigma_{r}-\sigma_{\theta }\right)-2G_{a}\left(\varepsilon_{r}-\varepsilon_{\theta }\right)}{2G\theta_{M}},$$

The relevant material parameters are characterized as a linear elastic response (3b), volume deformation (3c), linear bulk modulus (3d), linear Young’s modulus (3e), and linear shear modulus (3f). According to conventional considerations, it is assumed that Poisson’s ratio u is constant, and the elastic stage is independent of other strains and strain rates.

(3a) $$\frac{\partial \sigma }{\partial t}+\frac{\sigma }{\theta_{M}}=\left(E_{a}+E_{M}\right)\frac{\partial \varepsilon }{\partial t}+\frac{E_{a}\varepsilon }{\theta_{M}},$$

(3b) $$\sigma =E_{a}\varepsilon$$

(3c) $$\Delta =\varepsilon_{r}+2\varepsilon_{\theta }$$

(3d) $$K=\frac{E}{3\left(1-2\nu \right)}$$

(3e) $$E=E_{a}+E_{M}$$

(3f) $$G=\frac{E}{2\left(1+\nu \right)},$$

In this way, in order to describe the linear viscose-elastic spherical wave propagation problem, based on the standard linear solid constitutive relation, the governing equation reflecting the linear and high strain rate effect of materials is established. 

### 2.2. Experimental Method

In order to understand the propagation characteristics of spherical waves in concrete, an experimental method is developed to measure the particle velocity histories of spherical waves. The experiment adopts the electromagnetic method, and the sample is a cylinder with a diameter equal to the height. Because the arrangement of particle velocimeters have accurate representative characteristics, the method has strong advantages in studying the dynamic properties of multicomponent composites containing fillers, such as polymer–matrix composite materials, concrete, and rock in 3-D stress. In the spherical wave experiment, the characteristic size of the sample can be meters, which is more than ten times larger than the size of concrete coarse fillers, so that the information of wave histories can accurately reflect the wave propagation characteristics. A group of particle velocity waves v(r_i_,t) at different radii distance r_i_ from the center of the sphere is measured by a series of embedded magneto-electric velocimeters.

**Figure 2:**
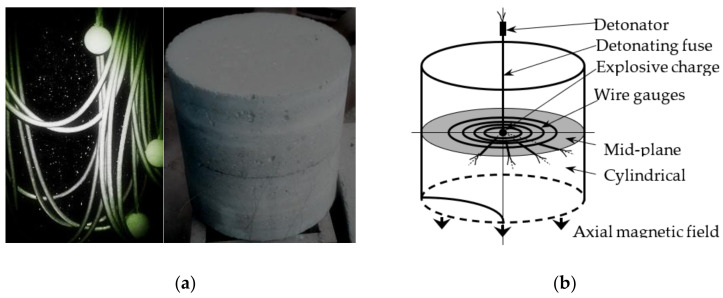
Scheme for velocity history test device in concrete with mini-charge: (**a**) mini-charge with soft detonating cord and long cylindrical block of concrete; (**b**) experimental concept for spherical wave experiments.

In the experiment, a mini-charge is detonated in the center of a cylindrical concrete block with a diameter of 25 cm and a length of 25 cm, and a spherical impact is loaded by detonating an explosive with a weight of 0.1 g/0.8 g. The principle of the spherical particle velocity history device is shown in Figure 2 [36]. The experimental specimen consists of two equal-height cylinder parts. A series of concentric toroidal magneto-electric particle string gauges is arranged on the mating surface. Explosive charges are placed in the cavity at the center of the sample; the soft detonating cord for initiation is entered along the mini hole of the upper half of the sample, and the upper and lower parts are bonded with epoxy resin after the gauge and the explosive charge are placed. After initiation, the particle velocimeters move to cut the magnetic field to form voltage signals, and the particle velocity histories at a series of radii can be obtained from the calibration results.

### 2.3. Inverse Method

The particle velocities in spherical wave propagation are easy to measure, but other physical quantities are difficult to measure directly at the same time. In order to obtain accurate information about other physical quantities during spherical wave propagation, and then obtain the constitutive relation of materials, Lagrangian inverse analysis is a good alternative, which is based on conservation equations and does not make any assumptions [37,38,39,40]. Next, the “second type inverse problem” in mathematics is dealt with to determine the dynamic constitutive properties of concrete. In the study of spherical waves, when the particle velocities at a series of different Lagrangian coordinates ri are obtained, it is difficult to calculate other unknown quantities from the former (2a, 2b). So we developed a new spherical wave analysis method “NV+T0/SW” to deal with this problem [14].

#### 2.3.1. The Method Solving Strain (ε_r_, ε_θ_)

The differential relation of strain (ε_r_, ε_θ_) and the particle velocity is established by the conservation equation. Now, the initial condition t = 0, v(r_i_,t)=0 is known, and v(r_i_,t) at different positions r_i_ (i = 1,2, …,) is also known. So, the time numerical integration operation can be performed to determine ε_θ_(r_i_, t). Then, the first derivative ∂v(r_i_,t)/∂t can be obtained by numerical differential operation. Similarly, the strain ε_r_(r_i_,t) can be determined by integrating time.

#### 2.3.2. The Method Solving Strain (σ_r_, σ_θ_)

However, the stresses σ_r_ and σ_θ_ are still unknown. The system composed of volume and shape deformation is to be determined. The solving of σ_r_ and σ_θ_ in this way is not sufficient, and one of the equation relations must be known first. In the elastic range, it is accepted that the volume deformation satisfies the linear law of elasticity (2a) and is independent of the rate. Then, it is easy to determine this relationship under quasi-static conditions. The calculation process related to quantity ε_r_ and ε_θ_, ∂σ_θ_/∂t, and ∂σ_r_/∂t can be expressed in Equations (4) and (5b).

In order to establish the magnitude relationship at each radius, the path-line processing method can be used to define the total derivative of a certain magnitude on the path-line (Grady, 1973), and the path-lines P1, P2, P3…Pi… Pm can be established as shown in the figure. In this way, when the spherical particle velocity histories v(r_i_,t) at multiple Lagrangian radii r=r_i_ are provided, and their related other time and position differential components ∂v(r_i_,t)/∂t can be easily determined.

(4) $$\frac{\partial \sigma_{\theta }}{\partial t}=\frac{1}{2}\left(\left(3K\frac{\partial \varepsilon_{r}}{\partial t}+2\frac{\partial \varepsilon_{\theta }}{\partial t}\right)-\frac{\partial \sigma_{r}}{\partial t}\right)$$

(5a) $${\left.\frac{d\sigma_{r}}{dr}\right|}_{p}={\left.\frac{\partial \sigma_{r}}{\partial r}\right|}_{t}+{\left.\frac{\partial \sigma_{r}}{\partial t}\right|}_{r}\frac{dt}{dr}{\left.={\left.\frac{\partial \sigma_{r}}{\partial r}\right|}_{t}+{\left.\frac{\partial \sigma_{r}}{\partial t}\right|}_{r}\frac{1}{r^{\prime }}\right|}_{p}$$

substituting (4) into (5a), the calculation formula of partial derivative about stress ∂σ_r_/∂t can be expressed as (5b).

(5b) $$\frac{\partial \sigma_{r}}{\partial t}=r^{\prime }\left({\left.\frac{d\sigma_{r}}{dr}\right|}_{p}-\rho_{0}\frac{\partial v}{\partial t}+\frac{2\left(\sigma_{r}-\sigma_{\theta }\right)}{r}\right)$$

**Figure 3:**
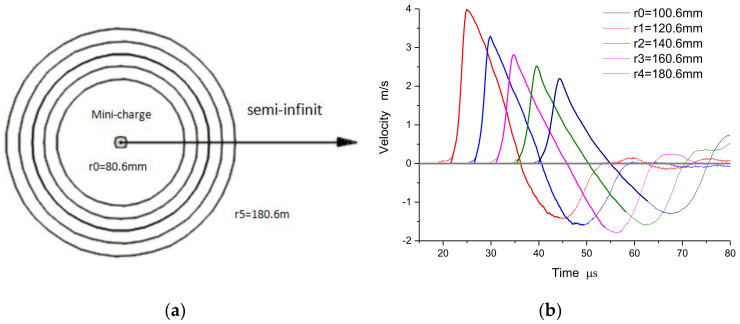
Results of velocity histories in concrete: (**a**) schematic diagram of test location layout in Mid-plane; (**b**) the series of measured particle velocity profiles.

The zero initial condition is known at different positions of wave propagation (σ_r_ = 0 along path-line P1), and the stress σ_r_ at different radius r=r_i_ along the path-line P2 (Figure 3) is obtained through the integration of partial derivative ∂σ_r_(r_i_,t)/∂t by using the constructed path-lines (5). Then, ∂σ_θ_(r_i_,t)/∂t is known from (4), and the circumferential stress at different positions r = r_i_ on the path-line P1 σ_θ_(r_i_,t)|_P = j_ can be calculated by integrating ∂σ_θ_(r_i_,t)/∂t. Similarly, the stress σ_r_(r_i_,t)|_P = j + 1_ and σ_θ_(r_i_,t)|_P = j + 1_ on all path-lines can be determined by cycling in sequence. Note that this method can be used to load the whole process, which is called “NV + T0/SW” for short.

#### 2.3.3. Solving for G and θ_M_

An advantage of “NV + T0/SW” is that there are no assumptions of the constitutive equation of materials, directly giving the stress–strain numerical relation. However, now there is a next step to take when the dynamic properties of materials are known. So, its description with a known standard linear solid constitutive model is provided in future work, and the dynamic shear modulus *G* and one Maxwell element material parameters *θ_M_* can be determined by the following method (6).

(6) $$G=\frac{\sigma_{r}-\sigma_{\theta }}{2\left(\varepsilon_{r}-\varepsilon_{\theta }\right)}$$

(7) $$\theta_{M}=\frac{\left(\sigma_{r}-\sigma_{\theta }\right)-2G_{a}\left(\varepsilon_{r}-\varepsilon_{\theta }\right)}{2G\left(\frac{\partial \varepsilon_{r}}{\partial t}-\frac{\partial \varepsilon_{\theta }}{\partial t}\right)-\left(\frac{\partial \sigma_{r}}{\partial t}-\frac{\partial \sigma_{\theta }}{\partial t}\right)}$$

## 3. Results

### 3.1. The Experimental Results 

Based on the experimental method as described in the previous section, the particle velocity profiles (Figure 3) in the low stress elastic region at the radii of 100.6 mm, 120.6 mm, 140.6 mm, 160 mm, and 180.6 mm are measured accurately in the semi-infinite space of concrete by using the mini-explosive ball and electromagnetic velocity measurement technology. Here, the radius of the mini-explosive ball is 5 mm, with an explosive equivalent of 1.00 g TNT. As shown in Figure 3b, the maximum particle velocity is lower than 4 m/s, and the experimental model is a one-dimensional spherical symmetry problem. At same time, the static mechanical property parameters of concrete can be easily measured, as shown in Table 1. 

### 3.2. The Inverse Numerical Results 

**Figure 4:**
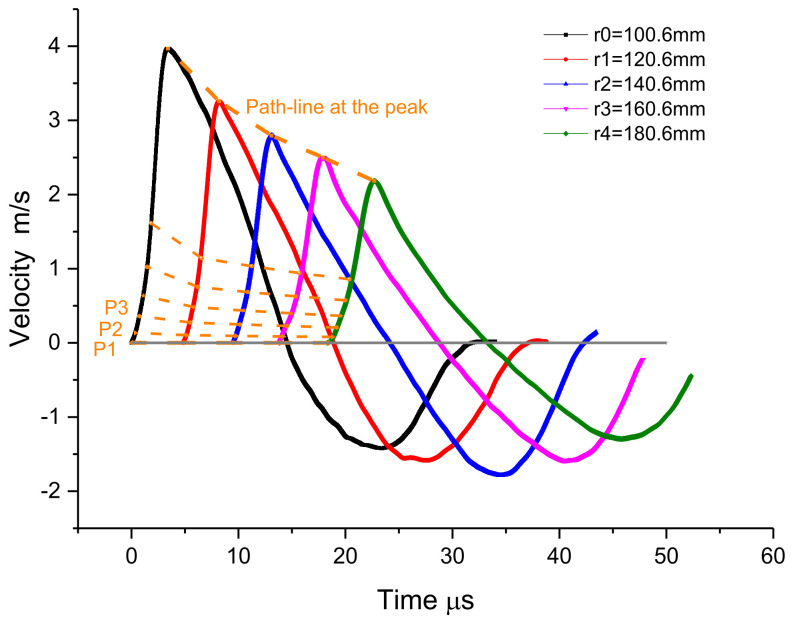
The schemes of inversion analysis with path-line.

**Figure 5:**
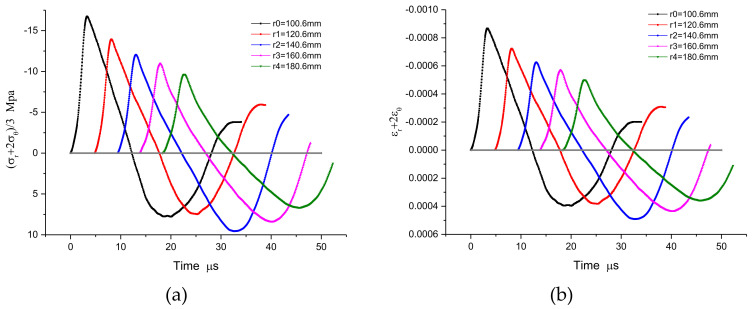
A comparison of positive and inverse results: (**a**) the volumetric part histories of stress σ_r_ + 2σ_θ_; (**b**) the volumetric part histories of strain ε_r_ + 2ε_θ._

**Figure 6:**
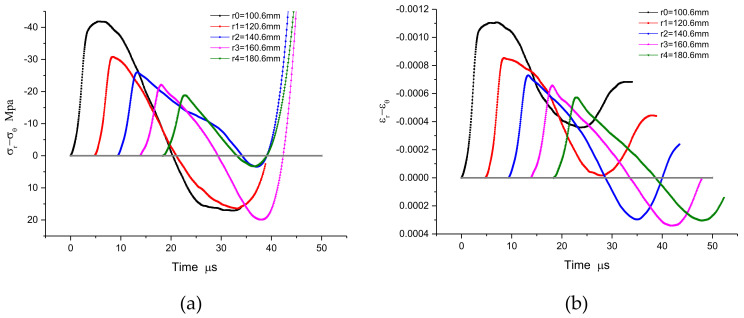
A comparison of positive and inverse results: (**a**) the distortional part histories of stress σ_r_ − σ_θ_; (**b**) the distortional part histories of strain ε_r_ − ε_θ._

**Figure 7:**
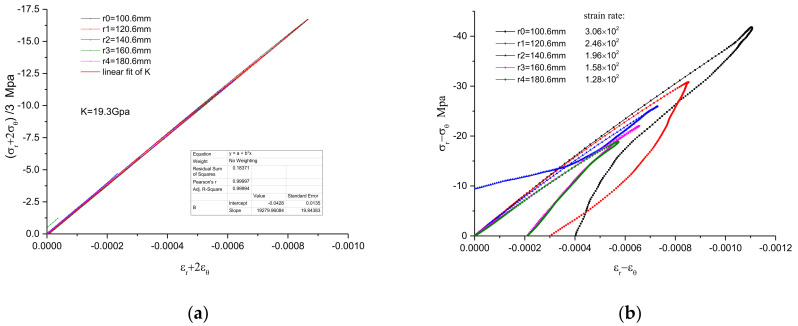
The numerical rate-dependent constitutive relation: (**a**) the volumetric relation of stress (σ_r_ + 2σ_θ_)/3 and strain ε_r_ + 2ε_θ_ stress σ_r_ − σ_θ_; (**b**) the distortional relation of stress σ_r_ − σ_θ_ and strain ε_r_ − ε_θ._

In the series of measured particle velocity histories shown in the Figure 4, the path-line is constructed from the initial zero value line. The path-line is divided into regions by the peak value. The analysis value of the path-line is interpolated at equal time intervals in each region to serve as the basis of the inversion analysis framework. With these path-line values covering the particle velocity field, the physical quantities of the spherical wave can easily be solved by the aforementioned method, i.e., “NV + T_0_/SW”. Since the constitutive relation of materials is often described by volume deformation and shape deformation with multidimensional stress state, it is convenient to reflect the stress characteristics under 3-D stress. The results are expressed as spherical profiles of volumetric part and distortional part, such as stress histories σ_r_ + 2σ_θ_, strain histories ε_r_ + 2ε_θ_, stress histories σ_r_ − σ_θ_, and strain histories ε_r_ − ε_θ_ .The numerical results are shown in Figure 5 and Figure 6, and the numerical constitutive relation, expressed in the form of volume and distortion, is shown in Figure 7. The volumetric constitutive relation satisfies linear elastic law with linear bulk modulus K, but the distortional constitutive relation does not. It is not difficult to find that the latter relation has an obvious rate effect.

### 3.3. The Determination of Dynamic Parameters G and θM

According to the above theory, the dynamic parameters G and θ_M_ can be determined from Equations (6) and (7), and the concrete static parameters and the numerical distortion relations of stress σ_r_ − σ_θ_ and strain ε_r_ − ε_θ_ are taken as the known conditions using the inverse method. The concrete static parameters used in the inverse analysis are the results of our experimental research on concrete under one-dimensional stress, and ρ and *ν* are measured from concrete samples, as shown in Table 1.

Note that Equation (6) is suitable for the series numerical distortion relations with different strain rates at each radius, so the average value of dynamic parameters G can be calculated easily with Equation (6), as shown in Table 2. Similarly, the dynamic parameters θ_M_ can be obtained through Equation (7), and the values of θ_M_ are also listed in Table 2. The results show that the dynamic shear modulus G is larger than the static modulus G_a_ and decreases with the reducing of strain rate (Figure 7b). At the same time, the dynamic relaxation time θ_M_ increases with a reducing strain rate and is in the magnitude range of 10^−6^ s.

## 4. Discussion

Firstly, a series of particle velocity histories of spherical waves in concrete is measured by magneto-electric velocimeters at each radius, which provides a basis for an experimental study on the dynamic properties of concrete in 3-D stress state under high strain rates. The particle velocimeter is a very thin ring coil, which is very suitable for measuring the physical quantities in spherical waves that change with the spherical radius. It is a good choice for measuring the signals of spherical waves for non-perspective materials, except for magnetic materials. Secondly, by analyzing the experimental data v(r_i_,t) of the spherical particle velocity wave of concrete, the Lagrangian “NV + T_0_/ SW” inverse analysis is carried out using the path-line method, and the wave propagation information of each physical quantity of the spherical wave is obtained. The numerical constitutive relation is expressed in the form of distortion and has an obvious rate effect. The results shown in Figure 7 demonstrate the obvious different behaviors of concrete between dynamic loading and static loading normally, and the strain rate effect of concrete cannot be ignored with the strain rate range of 10^2^ 1/s. The numerical constitutive relation is deduced directly from the measurements and analyses of wave propagation signals, which should be more appropriate for the coupled effects between wave propagation and rate dependency, are considered. Next, the rate-dependent dynamic parameters in concrete are determined by the standard linear solid model, which is a typical and useful model for analyzing stress relaxation and creep behaviors of viscoelastic solids. The results of dynamic parameters show that the dynamic shear modulus G is larger than the static modulus G_a_ and decreases with the reducing of strain rate (Figure 7b). Furthermore, the dynamic relaxation time θ_M_ increases with reducing strain rate and is in the magnitude range of 10^−6^ s. 

## 5. Conclusions

The goal of this research was to expand the knowledge about the possibilities of studying rate-dependent constitutive relation and the determination of dynamic parameters based on spherical waves in concrete. According to the former, the main conclusions drawn from the above results are as follows:

1. The series particle velocity of spherical waves in concrete specimens is measured by a magneto-electric velocimeter, which visually shows the propagation characteristics of spherical waves of particle velocity. It provides convenience for the interpretation of spherical wave information in concrete. At the same time, it also creates good support for the experimental study of constitutive relation of dynamic properties of strong impact in multidimensional stress state of concrete (inverse analysis).
2. The inverse problem is solved by the newly proposed “NV + T0/SW” Lagrangian analysis method, with the measured series velocity profiles as known conditions. The results provide a basis of a further study on how to determine accurately and effectively rate-dependent constitutive relation of concrete at high strain rate. When the numerical constitutive relation is expressed in the form of distortion, it is found that the distortion law has an obvious rate effect.
3. Based on a series of numerical constitutive relation with different strain rates at each radius, the rate-dependent dynamic parameters in concrete are determined by the standard linear solid constitutive model. The dynamic shear modulus G is larger than the static modulus and decreases with reducing strain rate. The dynamic relaxation time θ_M_ increases with the reducing strain rate and is in the magnitude range of 10^−6^ s.

It should be emphasized that, if more experimental data in the strain rate range and more continuous particle velocity profiles are measured through the improvement and development of experimental loading and data acquisition technology, the results obtained by this method will be enriched into a series. This method has good applicability, especially in the study of the dynamic behavior of multicomponent composite materials with large-size particle filler for the characteristic size of specimens in spherical wave experiments could be in the order of meters.

## Figures and Tables

**Table 1:** Concrete static parameters for ‘NV+T_0_/SW’.

Symbol	*ρ*	*υ*	C_K_	K	G_a_	E_a_
Units	kg/m^3^	1	m/s	GPa	GPa	GPa
Value	2380	0.23	4347	19.26	12.68	31.20

**Table 2:** Dynamic parameters by ‘NV+T_0_/SW’.

Symbol	2G	G	E	*θ*_M_
Units	GPa	GPa	GPa	μs
Value at r0	39.68	19.84	48.81	−1.14
Value at r1	39.51	19.75	48.60	−1.22
Value at r2	38.58	19.29	47.45	−1.41
Value at r3	35.90	17.95	44.16	−1.40
Value at r4	34.22	17.11	42.09	−1.42

## Data Availability

Not applicable.
